# Bovine bone particulates containing bone anabolic factors as a potential xenogenic bone graft substitute

**DOI:** 10.1186/s13018-019-1089-x

**Published:** 2019-02-20

**Authors:** David S. Musson, Ryan Gao, Maureen Watson, Jian-Ming Lin, Young-Eun Park, Donna Tuari, Karen E. Callon, Mark Zhu, Nicola Dalbeth, Dorit Naot, Jacob T. Munro, Jillian Cornish

**Affiliations:** 1 Department of Medicine, University of Auckland, Auckland, 1142 New Zealand; 2 Auckland City Hospital, Auckland District Health Board, Auckland, 1023 New Zealand; 3 Department of Surgery, University of Auckland, Auckland, 1142 New Zealand

**Keywords:** Xenogenic bone graft, Demineralised bone, Bovine bone, Osteoblast, Osteoclast

## Abstract

**Background:**

Alternative grafts are needed to improve the healing of bone non-union. Here, we assessed a bovine bone product which retains the inorganic and organic components of bone, as an alternative bone graft.

**Methods:**

Bovine bone matrix proteins (BBMPs) were isolated from bovine bone particulates (BBPs) and tested in vitro. Primary rat osteoblast viability, differentiation, and mineralisation were assessed with alamarBlue®, real-time PCR, and von Kossa staining assays, respectively. Osteoclast formation was assessed in primary murine bone marrow cultures with TRAP staining.

Human osteoblast growth and differentiation in the presence of BBPs was evaluated in 3D collagen gels in vitro using alamarBlue® and real-time PCR, respectively. The efficacy of BBPs as an alternative bone graft was tested in a rat critical-size calvarial defect model, with histology scored at 4 and 12 weeks post-surgery.

**Results:**

In vitro, the highest concentration of BBMPs increased mineral deposition five-fold compared to the untreated control group (*P* < 0.05); enhanced the expression of key osteoblast genes encoding for RUNX2, alkaline phosphatase, and osteocalcin (*P* < 0.05); and decreased osteoclast formation three-fold, compared to the untreated control group (*P* < 0.05).

However, the BBPs had no effect on primary human osteoblasts in vitro, and in vivo, no difference was found in healing between the BBP-treated group and the untreated control group.

**Conclusions:**

Overall, despite the positive effects of the BBMPs on the cells of the bone, the bovine bone product as a whole did not enhance bone healing. Finding a way to harness the positive effect of these BBMPs would provide a clear benefit for healing bone non-union.

## Background

Despite advances in modern fracture management, up to 5% of all fractures and 20% of high energy fractures fail to adequately heal, leading to non-union [1]. Furthermore, common elective orthopaedic procedures, such as spinal fusions, are associated with high non-union rates (< 35%) [2–5]. Currently, autologous bone is the gold standard graft material for encouraging bony union in elective spinal, oncological, and maxillofacial procedures. Whilst effective, autologous bone grafting is limited by the finite amount of bone that can be safely harvested, the need for a second surgical site, and significant donor site morbidity [6–9].

In order to circumvent the shortcomings of autologous bone grafting, allografts, bone graft substitutes, and growth factors that encourage bony growth and union are being sought by orthopaedic and maxillofacial surgeons. However, despite the high cost of allograft, its use is associated with unpredictable and suboptimal clinical results [10], whilst clinically applicable growth factors such as bone morphogenic proteins (BMP) are expensive and associated with adverse outcomes such as heterotopic ossification, malignancy, neurological deficits, retrograde ejaculation, and infection [11, 12].

A cheap and readily available alternative is xenograft bone particles, which are commonly used to fill bony defects in dental surgeries [13–16]. These are mostly composed of the inorganic, mineral component of bone, with the protein content removed, and act as an osteoconductive filler. However, it is well known that sequestered within the bone matrix are factors that are active in bone, that regulate bone remodelling, and potently stimulate bone formation [17–19]. This has led to allogenic-sourced demineralized bone matrix being employed clinically as a bone graft [20, 21], where the inorganic mineral component is removed.

We hypothesised that growth factors present within the bone matrix could enhance the performance of bovine bone particles used as bone grafts. Thus, we identified a bovine bone product, currently marketed as a nutraceutical, which retains both the inorganic calcium apatite component of the bone and approximately 20% *w*/*w* bovine bone proteins (MCH-Cal™, Waitaki Biosciences, New Zealand).

In order to assess the potential of this product as a bone graft, we isolated the protein component from the mineral, quantified a number of known bone active growth factors and bone matrix proteins, and tested the bioactivity of the protein component in well-established in vitro bone cell assays. We then tested the effect of the bovine bone product as a whole on human osteoblast activity in vitro and examined its ability to improve bone healing in a rodent critical-size bone defect model.

## Materials and methods

All protocols involving use of animals have been approved by the University of Auckland Animal Ethics Committee.

For human cells, written informed consent was obtained from all patients and the New Zealand Ministry of Health Northern Regional Ethics Committee approved the collection of samples.

### Bovine bone particulates (BBP)

MCH-Cal™ was obtained in a particulate form from Waitaki Biosciences, Christchurch, New Zealand, and sterilised by γ-irradiation at 15kgray. The MCH-Cal™ particulate size was approximately 75 μm.

### Bovine bone matrix protein (BBMP) extraction from BBP

MCH-Cal™ (5 g) was washed with 35 mL of 0.02 M Tris-HCl (pH 7.5) for 20 min then centrifuged at 4 °C. The precipitate was resuspended in 35 mL of 0.5 M ammonium EDTA in 0.02 M Tris-HCl (pH 7.5), stirred overnight, and then centrifuged at 4 °C. The precipitate was resuspended in 11 mL water and centrifuged. The supernatants were filtered through Whatman #4 filter paper and dialysed in SpectraPor tubing with a molecular weight cut-off of 3500 Da for 4 days against eight changes of distilled water, followed by 2 days against four changes of 0.02 M ammonium carbonate.

### Quantification of extracted BBMPs by immunoassay

In order to demonstrate that the BBMPs contained proteins usually found in bone, the amount of total transforming growth factor-β (TGF-β), insulin-like growth factor-1 and insulin-like growth factor-2 (IGF-1 and IGF-2), and osteocalcin was quantified. These proteins were selected as the levels of each of these have been previously quantified in bovine bone products [22]. TGF-β was assayed using Promega Emax® (Dade Behring) immunoassay kits, as per manufacturer’s instructions. Intact osteocalcin was assayed using an immunoassay kit (Metra Osteocalcin, Quidel Corporation, Santa Clara, CA). IGF-1 and IGF-2 were assayed using an in-house radioimmunoassay produced by AgResearch, NZ. Briefly, radiolabelled IGF-1 or IGF-2 and primary antibodies were incubated for 16 h at 4 °C. The primary antibodies for IGF-1 and IGF-2 are ProPep rabbit anti-human IGF-1 antiserum (Novozymes Biopharma AU Ltd.) and mouse anti-rat IGF-2 (Amano Enzymes USA Co., Troy, VA), respectively. Separation of bound and free antigen was achieved using a solid-phase second antibody-coated cellulose suspension. The secondary antibodies for IGF-1 and IGF-2 were SacCel® anti-rabbit IgG and SacCel® anti-mouse/rat IgG, respectively (IDS, Bolden, Tyne & Wear, UK). Following 30 min of incubation at 4 °C, bound antigen was sedimented by centrifugation and the soluble-free fraction removed by aspiration. Radioactivity in the pellet was counted in an LKB 1260 gamma counter (Wallac, Turkau, Finland). Calculations were performed using four-parameter logistics curve-fitting software.

All assays were carried out at least in duplicate.

### Primary osteoblast cell culture

Primary rat osteoblasts were isolated from E20 fetal rat calvariae, and primary human osteoblasts were grown from trabecular bone explants obtained from patients undergoing knee arthroplasty, as previously described [23, 24]. Briefly, trabecular bone explants were chopped into small bone chips and the bone marrow removed by repeated washes with 1× PBS. The bone chips were placed in T75 flasks with Dulbecco’s modified Eagle’s medium (DMEM) containing 10% fetal bovine serum (FBS) and 5 μg/ml L-ascorbic acid 2-phosphate (AA2P). When osteoblast outgrowth was first observed, the medium was refreshed and the outgrowing osteoblasts, having twice weekly media changes, were grown to 90% confluence.

For 3D cultures, 3 mg/mL rat collagen type I gels were used (BD Biosciences), as previously described [25]. Human osteoblasts suspended in culture medium were seeded in 50 μL collagen gels at 10^4^ cells per gel. Gels were allowed to set at 37 °C for 1 h before addition of DMEM + 10% (FBS) + 10μg/mL AA2P culture medium. BBPs were incorporated into the collagen gels at a concentration of 25 mg/mL.

### Cell viability assays

Primary rat osteoblasts were seeded into 24-well plates (2.5 × 10^4^ cells/well) for 24 h in MEM with 5% FBS and 5 μg/mL AA2P. Cultures were then growth-arrested in MEM with 0.1% bovine serum albumin (BSA) and 5 μg/mL AA2P for 24 h. Fresh growth arresting media and the BBMP solution (0, 20, 50, or 100 μL/mL) were added for a further 24 h.

In the 3D collagen gels, primary human osteoblasts were seeded as described above and cultured for a period of 1, 3, 7, and 14 days. Media were replaced every 2–3 days.

At each time point, alamarBlue® (Invitrogen) (5% final concentration in well) was added for 4 h at 37 °C. At the end of this incubation, 200 μL of the alamarBlue®-conditioned medium was transferred from each well to a 96-well plate and fluorescence (excitation 540 nm; emission 630 nm) read using a Synergy 2 multi-detection microplate reader (BioTek Instruments Inc., USA).

### Bone nodule assay of matrix deposition and mineralisation

Rat osteoblast cells were seeded into six-well plates at 5 × 10^4^ cells/well in αMEM with 10% FBS and 5 μg/mL AA2P. When confluent, media were changed to 15% FBS in αMEM supplemented with 50 μg/mL AA2P and 10 mM β-glycerophosphate, and a range of concentrations of the BBMPs (0, 20, 50, or 100 μL/mL) was added. These supplemented media were changed twice weekly, and fresh BBMPs were added. After 21 days, the cells were fixed in 10% neutral buffered formalin. Cultures were stained for mineral using von Kossa stain. The amount of mineralisation was quantified using BIOQUANT OSTEO software and reported as percentage area (BIOQUANT Image Analysis Corp., Nashville, TN).

### Gene expression analysis

RNA for real-time PCR was prepared from primary osteoblasts seeded as above and treated with the extracted proteins for 1, 3, and 7 days, as previously described [25].

Cells were lysed using RLT buffer with β-mercaptoethanol (QIAGEN Pty Ltd., VIC, Australia). Collagen gels were dissolved in RLT buffer with β-mercaptoethanol (QIAGEN Pty Ltd) and incubated at 55 °C with 0.2 mg/mL proteinase K (Life Technologies) for 15 min. Ethanol (70% *v*/*v*) was added and RNA extracted using the RNeasy mini kit (QIAGEN). Genomic DNA was removed from all RNA preparations with the RNase-free DNase set (QIAGEN). The quantity and purity of the RNA were measured using a NanoDrop Lite spectrophotometer (Thermo Scientific, Victoria, Australia), with a 260/280 absorbance value of > 1.8 being considered acceptable.

cDNA was synthesised from 500 ng of RNA with Superscript III (Life Technologies). Multiplex real-time PCR was carried out in the ABI PRISM® 7900HT Sequence Detection System (Life Technologies). Primers and probe sets were purchased as TaqMan® Gene Expression Assays (Life Technologies). All probes used to detect target genes were labelled with FAM™, and the 18S rRNA endogenous control probe was labelled with VIC®. The ΔΔCt method was used to calculate the relative levels of expression compared to a control sample from day 1 [26].

### Osteoclastogenesis assay

Bone marrow was obtained from the long bones of Swiss CD-1 male mice aged 4–6 weeks, as previously described [24, 27]. Bone marrow cells were incubated for 2 h, after which the non-adherent cells were collected and seeded in 48-well plates. Fresh medium and 10^− 8^ M 1,25-dihydroxyvitamin D3 was added on days 0, 2, and 4, and the BBMPs were added on days 2 and 4 of culture. Cultures were maintained for 7 days, at which time the cells were fixed, washed, and stained for tartrate-resistant acid phosphatase (TRAP) using the acid phosphatase, leukocyte kit (Sigma). Positive cells with three or more nuclei were recorded as osteoclasts. There were eight wells per test group, and the experiment was carried out three times with similar results.

### Rat calvarial defect model

Forty sexually mature, similarly aged, male Sprague-Dawley rats weighing more than 300 g were bred for this study. Pre-operatively, the rats were checked for general health, weighed, and randomised into two groups:

- Control group (*n* = 20): empty defects
- Experimental group (*n* = 20): BBP containing collagen gels

The surgical procedure was carried out as previously described [28]. Briefly, rats were pre-medicated with a non-steroidal anti-inflammatory analgesic, carprofen (10 μL/g) (Norbrook, New Zealand). Anaesthetic induction and maintenance was performed through a specialised nose cone (5% isoflurane with 2 L oxygen for induction and 2.5% isoflurane with 2 L oxygen for maintenance).

Once appropriately anaesthetized, an incision centred over the sagittal suture was made to the periosteum. The periosteum was divided and elevated as a single flap. A 5 mm diameter defect was created at the centre of the right parietal bone using a trephine burr (Komet Trephine, HenrySchein, New Zealand) set at 10,000 rpm. The trephine tip was cooled with saline throughout. The BBP containing collagen gels were implanted into the defect site in the test group, and the periosteal flap was sutured to the contralateral side using a 4/0 Monocryl suture (Amtech Medical, New Zealand). The collagen gel used in this study has previously been used as a delivery system for bone regeneration and been shown to have no effect by itself [28]. The skin was then closed using continuous subcuticular 4/0 Monocryl sutures. Following closure, 0.2 mL of marcain (1.25 mg/mL solution) was infiltrated around the surgical site for post-operative analgesia (Amtech Medical, New Zealand).

Post-operatively, carprofen (10 μL/g) and saline (2.5 mL) were administered subcutaneously twice daily for 48 h for analgesia and fluid replacement. The rats were weighed daily and monitored for signs of illness, infection, pain, or distress twice daily for the first two post-operative days. Following this, they were weighed and checked daily until post-operative day 7. They were then weighed and monitored weekly.

At the end of the experimental procedures, the rats were euthanised with CO_2_. The calvariae were excised and immediately fixed in 10% neutral buffered formalin and stored at 4 °C on a shaker. After 3 days, the calvariae were transferred to 70% ethanol for storage at 4 °C.

### Histological assessment

Specimens were decalcified in 10% formic acid for 1 week. After decalcification, the specimens were paraffin embedded in a Leica APS 300S auto processor. Histological sections (10 μm) were prepared from the mid-point of the defects in the coronal plane using a Leica Microtome RM 2145. Two sections per sample were placed on Leica ApexTM Superior adhesive slides and stained with haematoxylin and eosin (H&E). Digital images of the stained sections were obtained using the MetaSystems VSlide slide scanner and Zeiss Axio Imager Z2 fully motorised microscope (Zeiss, Germany). Images were assessed by three investigators, blinded to treatments, and scored on a scale of 1 to 10 based on the amount of new bone formed, quality of bone (woven vs lamellar), vascularization, and presence or lack of inflammation (summarised in Table 1).

**Table 1:** Histological scoring system used to grade bone healing

Score	Bone defect coverage	New bone type	Vascularisation	Inflammation
	Percentage of defect bridged by bone	Nature of new bone within defect	Presence of vascularisation within newly formed bone	Presence of inflammatory cells around newly formed bone
0	0%	No new bone	No evidence of neovascularisation	Abundant inflammation and evidence of encapsulation
1	1-24%	Predominantly woven	Few new vessels (<10)	Relatively few (10-50) inflammatory cells present
2	25-49%	Predominantly lamella remodelled	Abundant neovascularisation	No evidence of inflammatory cell presence
3	50-74%	-	-	-
4	75-100%	-	-	-

### Statistical analysis

Graph Pad Prism 6 software (Graph pad software, USA) was used for statistical analysis. Data were analysed using either one-way or two-way ANOVA, with post hoc Dunnett’s or Bonferroni’s tests, as denoted in the figure legends. A 5% significance level (*P* < 0.05) is used throughout.

## Results

### Quantitative analysis of factors present in BBMP

IGF-1, IGF-2, total TGF-β, and osteocalcin were all present in the BBP, and the concentrations are summarised in Table 2.

**Table 2:** Concentration of known growth factors and matrix proteins present within BBMP

Growth Factor	Concentrations (μg/gram)
IGF-1	0.61
IGF-2	0.25
TGFβ (total)	0.19
Osteocalcin	595

### BBMPs decrease osteoblast viability and increase osteoblast differentiation

**Fig. 1:**
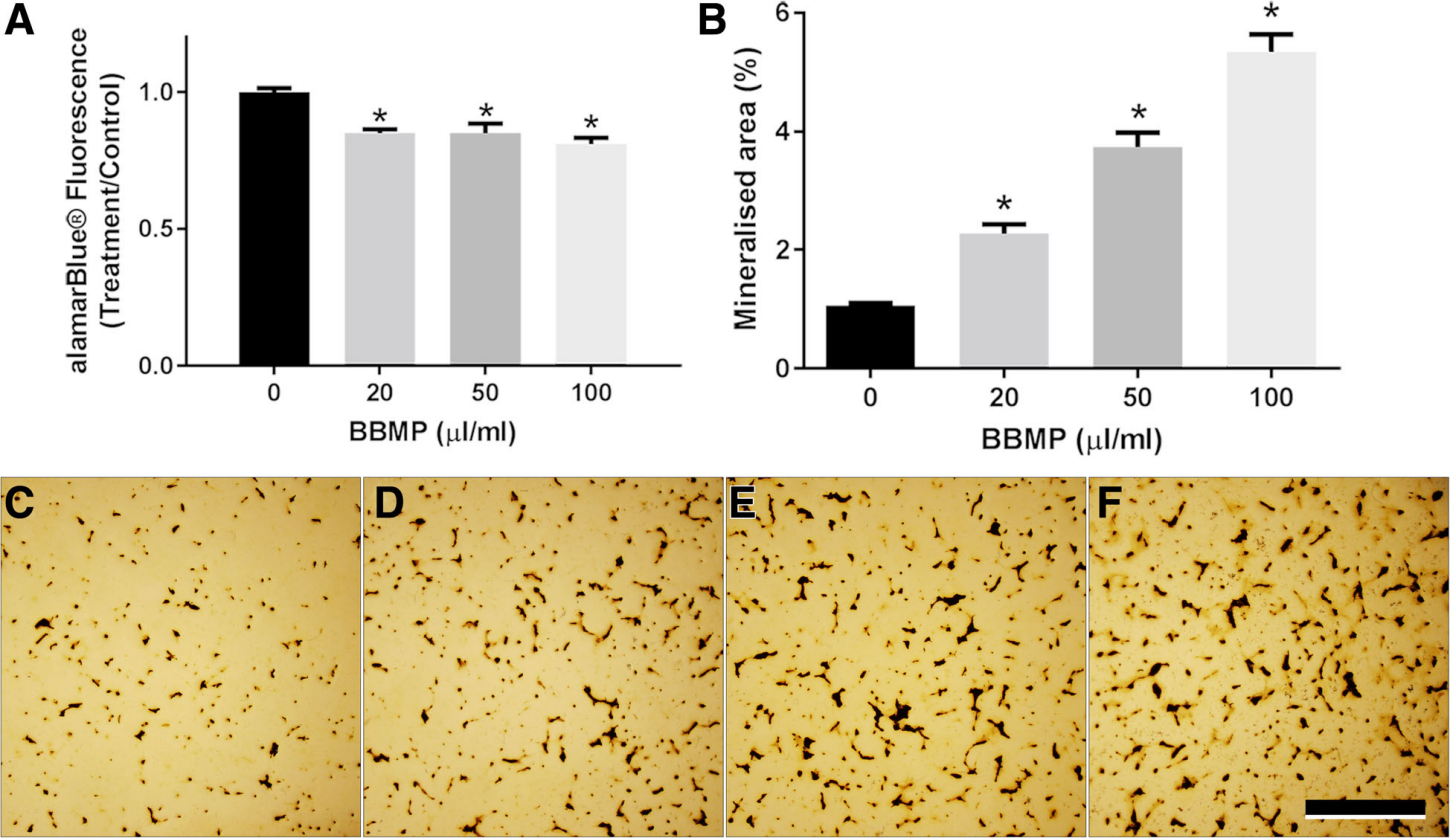
BBMP decreases osteoblast viability and dose-dependently increases osteoblast mineralisation. Effects of increasing concentrations of BBMP on (**a**) primary rat osteoblast viability, as determined by alamarBlue® assay and (**b**) von Kossa staining of bone nodules in primary rat osteoblasts cultured for 21 days. Data presented are the means of three biological experiments ± SEM. One-way ANOVA with post hoc Dunnett. *Significantly different from control (*P* < 0.05). (**c**–**f**) Representative images of von Kossa stained osteoblasts following 21-day culture with BBMP

Osteoblast viability was significantly reduced by all concentrations of BBMP after 24 h treatment (Fig. 1a). The greatest reduction in osteoblast viability was seen with 100 μL/mL BBMP (19%, *P* < 0.05), which was the highest concentration tested.

In a 21-day differentiation assay, all concentrations of BBMP significantly and dose-dependently increased the amount of osteoblast mineralisation (Fig. 1b), with 100 μL/mL BBMP increasing mineralised area five-fold compared to untreated control (*P* < 0.05).

**Fig. 2:**
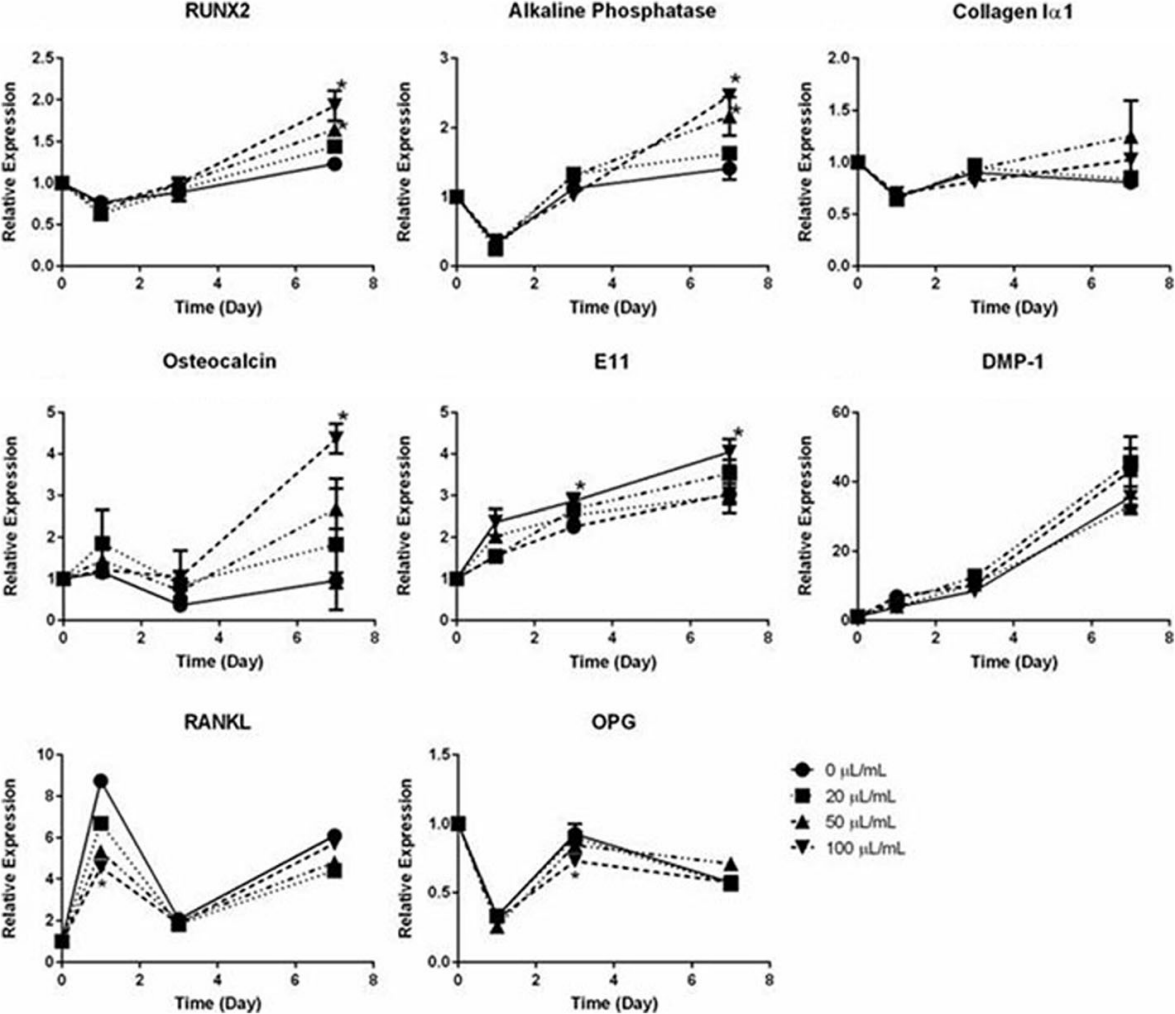
BBMP increases the expression of osteoblast marker genes. Effects of increasing concentrations of BBMP on the expression of osteoblast-related genes over a 7-day culture period. Data presented are the means of three biological experiments ± SEM. Two-way ANOVA with post hoc Bonforroni. *significantly different from control (*P* < 0.05)

Analysis of osteoblast marker gene expression showed that the highest concentration of BBMP (100 μL/mL) increased the expression of RUNX2, alkaline phosphatase, osteocalcin, and E11 after 7 days of treatment (all *P* < 0.05), but had no effect on dentin matrix acidic phosphoprotein 1 (DMP1) and collagen I expression. BBMP also had no effect on the expression of receptor activator of nuclear factor kappa-Β ligand (RANKL) and osteoprotegrin (OPG), genes that regulate osteoclast formation and function (Fig. 2).

### BBMPs inhibit osteoclast formation

**Fig. 3:**
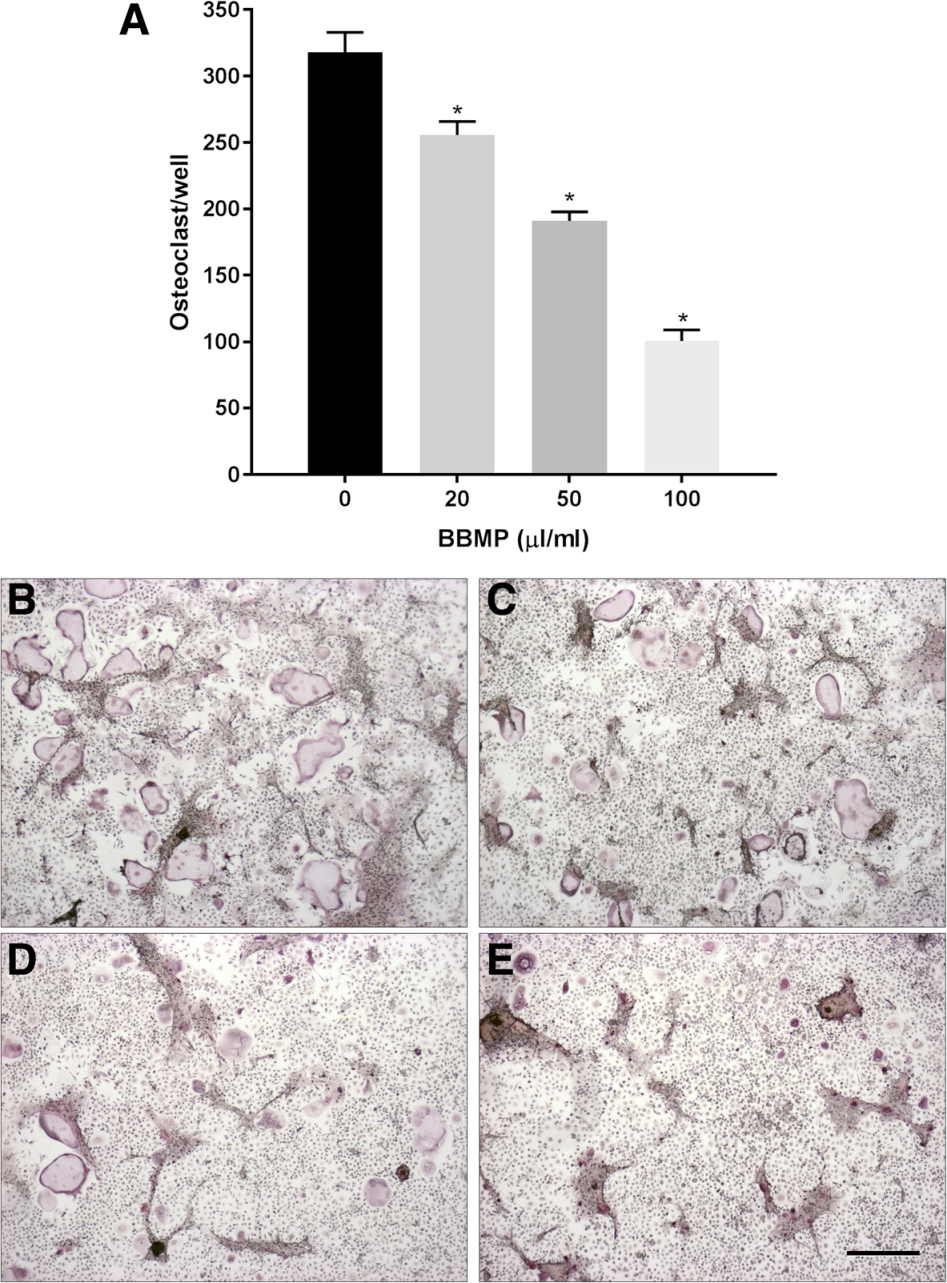
BBMP inhibits osteoclastogenesis in mouse bone marrow cultures. (**a**) Effects of increasing concentrations of BBMP on the formation of osteoclasts, as determined by the number of TRAP +ve multinucleated cells per well. Data are presented as means ± SEM. n = 4. One-way ANOVA with post hoc Dunnett’s. *Significantly different from control (*P* < 0.05). (**b**-**e**) Representative images of TRAP-stained cultures treated with BBMP are presented

All concentrations of BBMP significantly decreased the number of TRAP-positive multinucleated cells in a dose-dependent manner (Fig. 3, all *P* < 0.05). The highest concentration of BBMP (100 μL/mL) decreased osteoclast formation three-fold when compared to untreated controls.

### BBPs are cytocompatible with human osteoblasts

Having determined the activity of the organic protein component of the bovine bone particulates, the cytocompatability of the whole particulates was assessed in vitro with primary human osteoblasts.

**Fig. 4:**
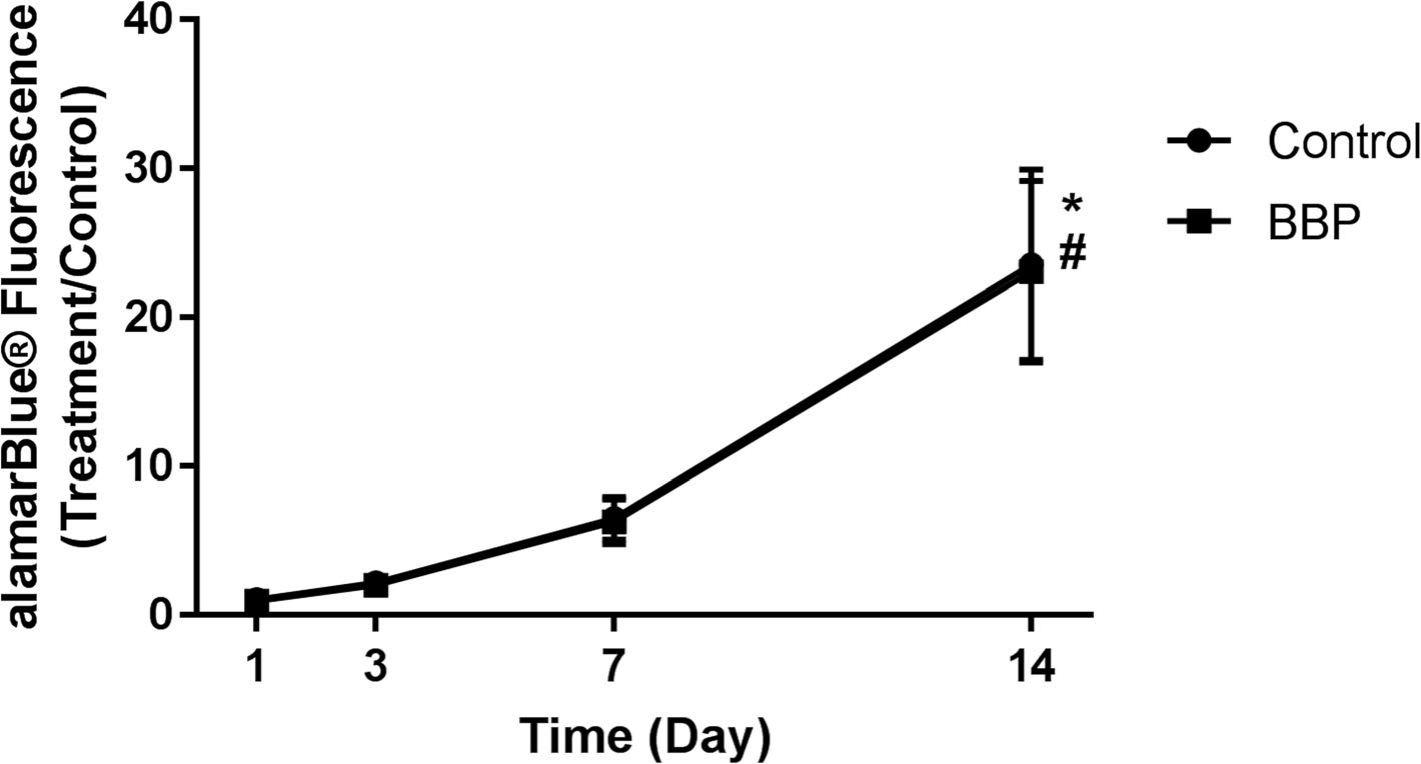
BBPs are cyto-compatible with human osteoblasts. Effects of BBP on primary human osteoblast growth in a collagen gel over 14 days of culture, as determined by alamarBlue® assay. Data presented are the means of three biological experiments ± SEM. Two-way ANOVA with post hoc Bonforroni. *Control group significantly different from day 1 (*P* < 0.05). ^#^Treatment group significantly different from day 1 (*P* < 0.05)

Assessment of cell viability over a 14-day culture period demonstrated an overall increase in the number of viable cells over time, with a significantly higher viability observed at day 14 in both groups compared to their respective viability at day 1 (*P* < 0.0001). However, there was no difference in the rate of growth between cells in collagen gels or in gels containing BBPs (Fig. 4).

### Collagen Iα1 and RANKL gene expression are reduced in the presence of BBPs

**Fig. 5:**
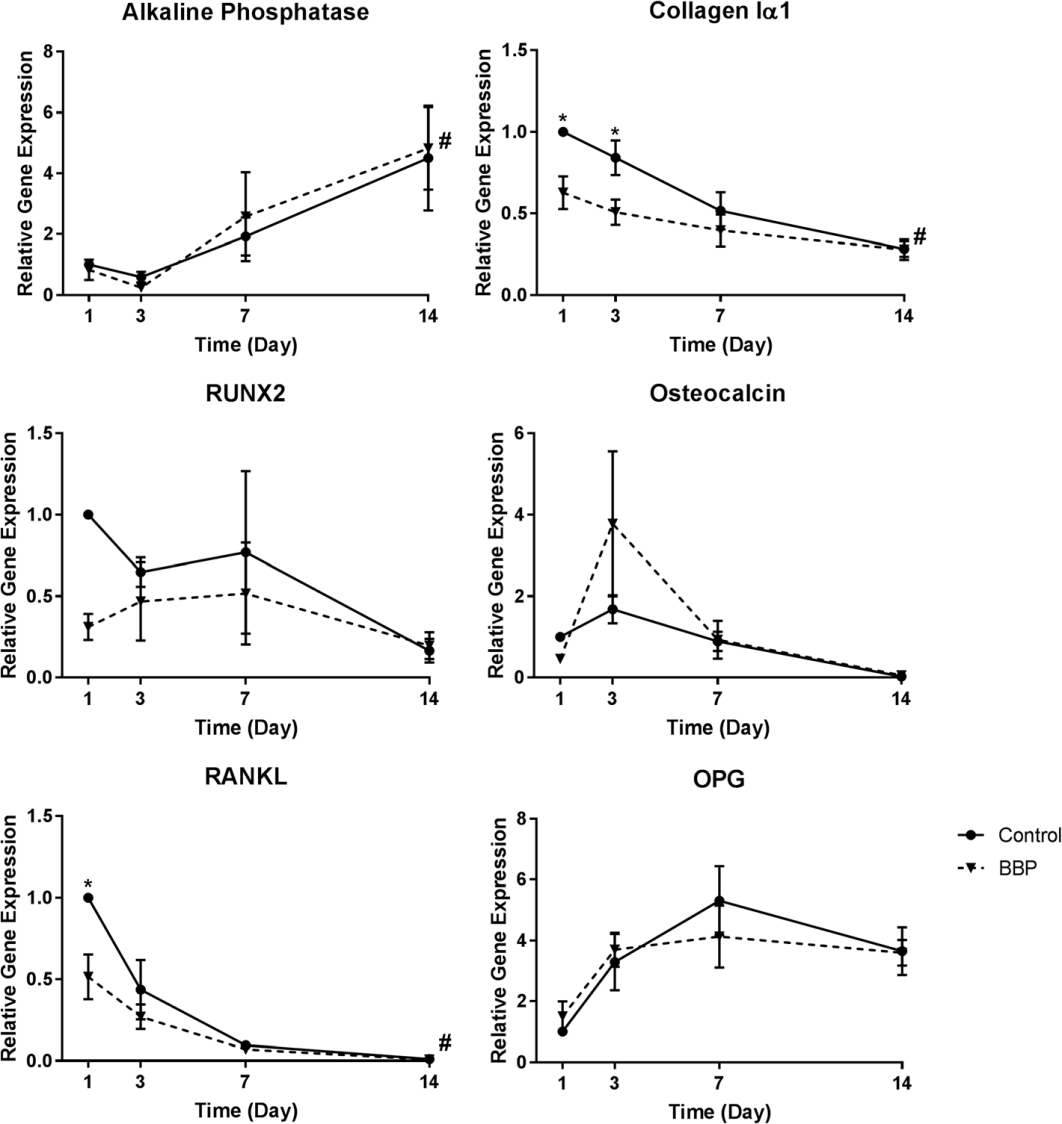
Collagen Iα1 and RANKL gene expression are reduced in the presence of BBPs. Effects of BBP on the expression of osteoblast-related genes in primary human osteoblast cells over a 14-day culture period. Data presented are the means of three biological experiments ± SEM. Two-way ANOVA with post hoc Bonforroni. *Significantly different from treatment (*P* < 0.05). ^#^Significantly different from respective group day 1 (*P* < 0.05)

The expression of osteoblast marker genes was determined over 14 days of primary human osteoblast culture in collagen gels with or without BBPs (Fig. 5). The expression of alkaline phosphatase significantly increased over the 14-day culture period in both groups (*P* < 0.05), whilst collagen type 1 alpha 1 (*P* < 0.05) and RANKL (*P* < 0.05) expression decreased over time. The expression of collagen type 1 alpha 1 was significantly reduced in the BBP-treated group after 1 and 3 days of culture, compared to the control groups, as was RANKL expression on day 1 (*P* < 0.05).

### In vivo, BBPs do not improve bone healing in a rat calvarial defect

**Fig. 6:**
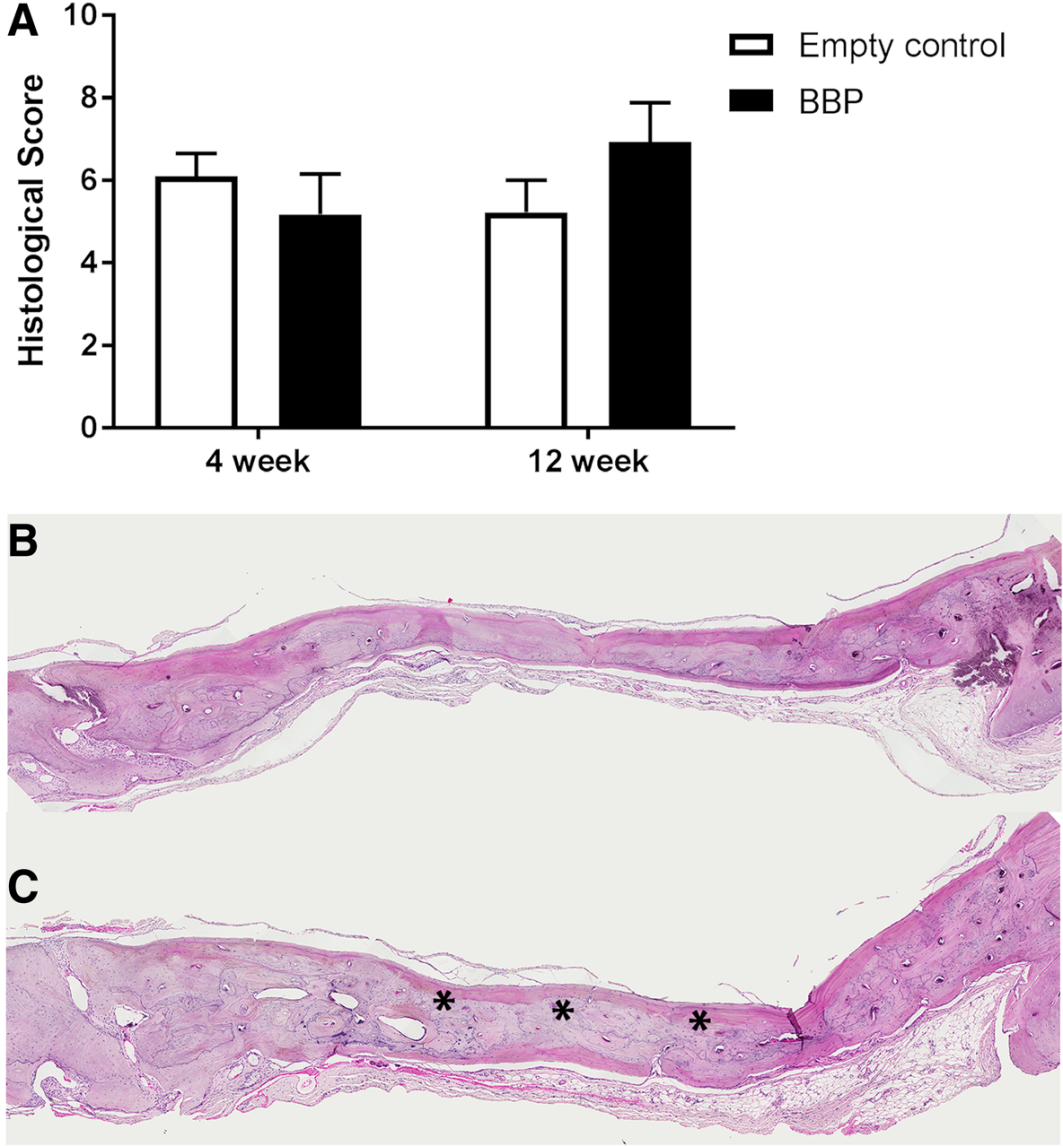
BBPs have no effect on bone healing in a rat calvarial defect. (**a**) Histological grading of the two groups at 4 and 12 weeks. Representative images of H&E stained coronal sections taken at the mid-point of the defects from the control group (**b**) and the BBP-treated group (**c**) 12 weeks post-surgery. Asterisk indicates the presence of BBP remaining within the bone matrix

The ability of the BBPs to improve bone healing was assessed in a rat critical-sized calvarial defect. Semi-quantitative histological assessment of healing at 4 and 12 weeks post-surgery by three researchers blinded to treatments identified a similar amount of new bone formation in the control group and the group treated with BBPs, with an even mix of woven and lamellar bone identifiable in both groups (Fig. 6a). Some particulates appeared to remain in the test group, although these had been incorporated into the new bone and there was no evidence of an adverse inflammatory response (Fig. 6c).

## Discussion

Here, we have shown that the bovine bone matrix proteins (BBMPs) isolated from bovine bone particulates (BBPs) were osteoinductive, demonstrating potent anabolic and anti-catabolic activity in bone cells in vitro. Despite this, the BBPs themselves did not offer any osteoinductive activity in our models in vitro or in vivo. This is the first study to confirm the bioactivity of BBMPs on osteoblasts and osteoclasts in vitro and provides support for the use of xenogenic factors for improving the outcomes of orthopaedic surgeries.

The osteogenic effect of matrix proteins sequestered within the bone was first described in the seminal work of Urist, who implanted decalcified or demineralised bone into the muscle bed of small animals and observed potent ectopic bone formation [29]. This led to the term bone morphogenetic protein (BMP) being used to describe the unknown factor present within the bone matrix that induced the observed osteogenic effect [30]. It is now known that there are over 20 BMPs, which have various roles in tissue development and repair [17].

In addition to the BMPs, a number of other factors sequestered within the bone matrix have constitutive actions on bone, such as IGF-I and IGF-II, TGF-β1 and TGF-β2, fibroblast growth factors (FGFs), and platelet-derived growth factor (PDGF) [19]. In bovine bone, the presence of TGF-β1 and TGF-β2 and IGF-1 was first described in 1988 [18], whilst the amounts of total TGF-β, IGF-1 and IGF-2, and osteocalcin were successfully quantified in a bovine bone product in 1991 [22]. Here, we have found similar levels of these factors to be present within our BBMPs. The individual effects of these factors on bone cells are well established, with TGF-β known to inhibit osteoclast formation and activity [31], and the majority of factors present being capable of inducing osteoblast growth and differentiation [32].

Given the positive effects of the BBMP in vitro, it was expected that a similarly positive effect would be seen with the BBPs as a whole. However, this was not the case. The absence of a positive response to BBP is likely due to a lack of availability of the positive factors within the particulates. In bone remodelling, these factors are released during bone resorption, as the osteoclasts degrade the mineral and collagenous matrix [33]. There is some evidence that despite the presence of osteoclasts on the surface of xenograft particles in bone defects, they do not get actively resorbed. Instead, the particulates become surrounded by the newly formed bone [34, 35]. Similar effects could be occurring with the BBPs in our in vivo study, as the particulates are still present within the newly formed bone after 12 weeks.

Whilst our studies have attempted to utilise an extensive combination of in vitro and in vivo techniques to assess the BBPs as a novel bone graft, a limitation of our study is that pre-clinical models will never fully replicate the clinical setting. The use of animal cells and models is removed from humans, and therefore, translation to a clinical setting is problematic. To address this, we also used cells isolated from human tissues. In keeping with previous studies [23, 36], here, we used samples come from both male and female patients undergoing knee arthroplasty and include a range of ages. This is both a strength and limitation as it encapsulates a broader population of patients, but increases variability. In order to account for this, we carry out multiple biological repeats of our experiments using samples from different patients. Reassuringly, the multiple biological repeats in our study had similar results, suggesting that our findings are translatable.

In summary, we have shown that the proteins present within bovine bone particulates are both anabolic and anti-catabolic to bone cells, but when left within the particulates, they do not offer any additional effects above the osteoconductive nature of the xenograft BBP. Modifying the preparation of the particulate material to allow for the release the bone-active factors into the microenvironment would likely improve the overall performance of the grafts. Similarly, using this product as a basis for the production of a xeno-demineralised bone matrix paste could offer an easily sourced, cheaper bone graft alternative to allograft demineralized bone matrix, which is estimated to be used successfully in 20% of all bone grafting procedures and only limited by the high cost of the material.

## Conclusions

Based on the work presented here, in its current form, these BBPs offer a purely osteoconductive material, with no anabolic advantage likely to be provided by the abundant growth factors present within the particulates. However, given these factors clearly produce an anabolic bone effect, finding a way to harness this either through a change in preparation of the material or through the production of a xeno-demineralised bone matrix paste could provide a clear benefit for surgeons faced with large bony defects, which left by themselves fail to heal and reduce the integrity of the surrounding bone.

## Abbreviations

AA2P: L-ascorbic acid 2-phosphate
BBMPs: Bovine bone matrix proteins
BBPs: Bovine bone particulates
BMP: Bone morphogenic protein
BSA: Bovine serum albumin
DMEM: Dulbecco’s modified eagle’s medium
DMP1: Dentin matrix acidic phosphoprotein 1
FBS: Fetal bovine serum
FGF: Fibroblast growth factor
H&E: Haematoxylin and eosin
IGF-1: Insulin-like growth factor-1
IGF-2: Insulin-like growth factor-2
OPG: Osteoprotegrin
PBS: Phosphate buffered saline
PDGF: Platelet-derived growth factor
RANKL: Receptor activator of nuclear factor kappa-Β ligand
TGF-β: Transforming growth factor-β
TRAP: Tartrate-resistant acid phosphatase
